# Biophysical properties of the aorta in patients with Marfan syndrome and related connective tissue disorders: evaluation with MRI and computational fluid dynamics modeling

**DOI:** 10.1186/1532-429X-13-S1-P219

**Published:** 2011-02-02

**Authors:** Joseph A Camarda, Michael G Earing, Ronak J Dholakia, Hongfeng Wang, Sung Kwon, John F LaDisa, Margaret M Samyn

**Affiliations:** 1 Medical College of Wisconsin/Children's Hospital of Wisconsin, Milwaukee, WI, USA; 2 Marquette University, Milwaukee, WI, USA

## Objective

The purpose of this study was to evaluate the biophysical properties of the aorta in patients with Marfan Syndrome or Loeys-Dietz Syndrome using MRI and computational fluid dynamics (CFD) modeling.

## Background

Patients with Marfan Syndrome and related disorders have abnormal wall properties throughout the thoracic aorta including low distensibility in areas that eventually dilate. CFD modeling based on MRI data can be used to quantify distensibility and indices such as velocity and wall shear stress (WSS) throughout the cardiac cycle and for the entire thoracic aorta. We sought to use cardiac MRI and CFD modeling to evaluate the distensibility and indices of WSS in the aorta of patients with Marfan Syndrome and related disorders compared to age- and gender-matched controls.

## Methods

Patients with Marfan Syndrome or a related disorder and no history of surgery who underwent a recent MRI were identified (n=6; 5 Marfan, 1 Loeys-Dietz). After measuring blood pressure, aortic wall dimensions and distensibility were calculated off-line by two independent observers at 3 locations: ascending aorta (AAo), thoracic descending aorta (DAoT), and descending aorta at the level of the diaphragm (DAoD). CFD simulations were performed using a stabilized finite element solver and incorporated downstream vascular resistance and compliance CFD models to quantify time-averaged WSS (TAWSS) and oscillatory shear index (OSI) throughout the aorta.

## Results

For the disease cohort, the greatest systolic area (944mm^2^) and diastolic aortic area (780mm^2^) were at the level of the AAo. Mean systolic and diastolic area differences between observers were 78.8mm^2^ and 47.3mm^2^, respectively. The lowest distensibility was at the level of the AAo, mean 4.73x10^-3^ Hg^-1^. The total vessel wall surface area exposed to sub-normal TAWSS (<15 dyn/cm^2^) was similar for both the Marfan and control groups (65.9% vs. 56.4%, respectively). However, the surface area exposed to elevated OSI (>0.15) was greater in the Marfan and related group than controls (53.2% vs. 39%, respectively) and most pronounced in the AAo.

## Conclusion

In this small cohort**,** aortic wall distensibility is lowest at the level of the AAo and CFD modeling revealed this area also contains abnormal TAWSS and OSI as compared to control patients. Interestingly, this area often undergoes progressive dilation requiring aortic root replacement surgery. CFD modeling, therefore, may be used in concert with MRI to predict areas of future dilation, but further studies are required to evaluate the relationship of these findings and the clinical course.

